# Recognition of Facial Expressions in Individuals with Elevated Levels of Depressive Symptoms: An Eye-Movement Study

**DOI:** 10.1155/2012/249030

**Published:** 2012-01-11

**Authors:** Lingdan Wu, Jie Pu, John J. B. Allen, Paul Pauli

**Affiliations:** ^1^Departments of Psychology, Biological Psychology, Clinical Psychology, and Psychotherapy, University of Würzburg, Marcusstraße 9-11, 97070 Würzburg, Germany; ^2^Department of Psychology, University of Arizona, Tucson, AZ 85721, USA

## Abstract

Previous studies consistently reported abnormal recognition of facial expressions in depression. However, it is still not clear whether this abnormality is due to an enhanced or impaired ability to recognize facial expressions, and what underlying cognitive systems are involved. The present study aimed to examine how individuals with elevated levels of depressive symptoms differ from controls on facial expression recognition and to assess attention and information processing using eye tracking. Forty participants (18 with elevated depressive symptoms) were instructed to label facial expressions depicting one of seven emotions. Results showed that the high-depression group, in comparison with the low-depression group, recognized facial expressions faster and with comparable accuracy. Furthermore, the high-depression group demonstrated greater leftwards attention bias which has been argued to be an indicator of hyperactivation of right hemisphere during facial expression recognition.

## 1. Introduction

Facial expression is the most powerful, natural, and direct way to communicate emotion in everyday social interaction [1]. Individuals detect emotional feedback and behave reciprocally on the basis of identifying others' facial expressions, which has important adaptive value to humans [2]. Depression, however, has been associated with negative schema regarding the self, external events, and situations as suggested by cognitive theories of depression [3, 4]. In particular, depressed participants are proposed to be alert to negative information and to engage in continuous negative-feedback seeking during social interaction [4]. Accordingly, it is generally assumed that depressed individuals may show abnormal patterns in recognition of facial expression. 

Numerous studies have noted differences between depressed and healthy individuals during recognition of facial expressions. However, it is still not clear whether depression is associated with an enhanced or impaired ability to recognize facial expressions. Some studies reported that individuals with depressive symptoms are more accurate than healthy individuals in recognizing both negative and positive facial expressions, suggesting a global hypervigilance to emotional facial expressions [5, 6], while others reported that depression is associated with either specific deficit in recognizing facial expressions: happiness [7], sadness [8, 9], anger [10], or a global deficit in recognizing both positive and negative facial emotions [10–12].

In part, this controversy can be attributed to the fact that used tasks in those studies (e.g., visual searching task and picture matching task) differ in their capability to differentiate individuals' ability to recognize emotional facial expressions. For the visual searching task, it has been demonstrated that the detection of a target facial expression is affected by the number of distracters, context, and searching strategies [13, 14]. For the picture-matching task, the same-different judgment on two facial expressions can be performed by matching facial features without extracting emotional cues from the expressions, and therefore this task did not differentiate the ability to recognize the emotion of facial expressions [7, 15, 16]. A comparably more direct and more sensitive task is the verbal labeling task. Unlike visual searching and picture-matching tasks, the verbal labeling task requires participants to name the emotion of each facial expression and therefore forces the participant to rely more on emotional information [15].

Another possible reason for the inconsistent findings may be due to the tradeoff between response time and response accuracy. It is important to consider the competing demands of speed and accuracy since individuals could either recognize facial expressions quickly with a larger number of errors or slowly with few errors. However, with only a few exceptions [5, 6], previous studies investigated response time without a report of response accuracy, or vice versa. To provide more convincing evidence for abnormal facial expression recognition in depressed individuals, further studies utilizing more sensitive tasks such as the verbal labeling task, in addition to comparing both response time and response accuracy during recognition of facial expressions, are required.

Although previous studies consistently reported differences between depressed and control groups in recognition of facial expressions, little is known about the cognitive systems underlying these differences. This is a key piece of missing information because it can help to understand and explain the affective and behavioral abnormalities in depression and hence offers practical implications for treatment.

Some recent studies applied eye-tracking technology which offers an objective, continuous, and noninvasive measure of when and where people look to investigate the cognitive systems underlying facial expression recognition. It has been found that facial features including eyes, mouths, nose, and eyebrows provide key clues for recognizing facial expressions [17, 18], and healthy individuals focus on those regions of interest (ROI) in order to recognize facial expressions. In addition, healthy individuals show left-biased eye movements [19], meaning that they spent more time on viewing the facial stimuli in the left visual hemifield. This bias is interpreted as an indicator of right hemisphere involvement in processing of facial expressions [19]. Individuals with anxiety disorder and schizophrenia were found to exhibit reduced dwell time on ROI and hence showed irregular scan paths during viewing of facial expressions [20]. So far, the eye-movement pattern during recognition of facial expressions in depression has not been studied. 

The present study applied eye-tracking technology to investigate whether and how individuals with elevated depressive symptoms differ from those with low depressive symptoms on facial expression recognition. We recorded continuous eye movements, as well as recognition accuracy and response time during a verbal labeling task. The specific aims of the present study are (1) to examine whether response speed and accuracy for facial expressions are altered by depression; (2) to examine whether depression has valence-specific effects on facial recognition in terms of selectively impacting recognition of positive versus negative facial expressions; (3) to investigate whether depression is characterized by altered eye-scan pattern.

## 2. Method

### 2.1. Participants

Participants were recruited through internet advertisements. A total of 48 undergraduate students (mean age = 21.55 years, SD = 1.62) took part in the study for either class credit or money. Data from 8 participants were unavailable due to data missing caused by problems with the eye-tracking apparatus. Of the remaining participants, 18 participants (9 females) scored greater than 50 on the self-rating depression scale (SDS) (M = 56.55, SD = 1.93) and were thus defined as the high-depression group (HD group). The other 22 participants (11 female) scored less than 50 on the SDS (M = 44.05, SD = 3.75) and served as the low-depression group (LD group). All participants had normal or corrected to normal vision and reported no history of clinically diagnosed psychopathology, speech disorders, or prior experience with this study.

### 2.2. Materials

#### 2.2.1. Self-Rating Depression Scale

The self-rating depression scale (SDS) consists of 20 items presented in a 4-point multiple-choice format. Individual items are scored between 1 and 4, with higher scores indicating increased level of depressive symptoms. The score ranges from 20–80 with 20–49 indicating normal range; 50–59 for mildly depressed; 60–69 for moderately depressed; 70 or above for severely depressed [21, 22]. In the current study, the SDS scores of individuals with elevated depressive symptoms ranged from 50 to 59.

#### 2.2.2. Chinese Affective Face Picture System (CAFPS)

CAFPS is a standardized and validated set of Chinese facial expression photographs which has been applied in studies in Chinese participants [23]. All pictures were taken with models looking straight ahead with different types of expressions (neutral and six basic facial emotions: happy, surprise, disgust, sad, fear, and angry), are in black-and-white and are described by means of intensity scores ranging from 1 to 9 (1 = low intensity and easy to recognize, 9 = high intensity and hard to recognize). Twenty-eight emotional face photos, each portraying one of the seven emotions, were selected from CAFPS. The average intensity score of these face photos was 6.60 (SD = 0.18). Both genders were equally represented in each of the seven categories of facial expressions. A different set of fourteen photos were selected for practice trials. Each picture was presented with a picture size of 700 × 600 pixels at a viewing distance of 60 cm.

### 2.3. Apparatus and Procedure

Stimuli were presented using the E-Prime software package (Psychology Software Tools, Inc; Pittsburgh). The start of each trial (except for the first one) was triggered by participants' verbal response recorded through a voice response box and therefore trials were self-paced. Eye movements were recorded using the head-mounted Eyelink-II (SR Research Ltd., Mississauga), with a sampling resolution of 500 Hz and a spatial resolution of 0.2°. An eye movement was classified as a saccade when its distance exceeded 0.2° and velocity reached 30°/s; a fixation was defined as consecutive gaze confined within a diameter of 1° visual angles, for at least 200 ms [18, 20]. A chin rest was used to immobilize participant's head during the experiment and to ensure a 60 cm distance between the center of the 17′′ computer screen and participant's eyes. A microphone connected with a voice response box was fixed 5 cm in front of the chin rest, at the same height.

Prior to the experiment, all participants gave their informed consent in accordance with Institutional Ethical Review Boards' procedure and requirements. They were then asked to memorize the seven verbal labels of facial expressions (neutral, happy, surprise, disgust, sad, fear, and angry) and repeat the labels as many times as possible until they could recall the seven labels without any effort.

The study was conducted in a quiet and darkened room. After an introduction of the experimental procedure, participants were equipped with the head-mounted EyelinkII and were instructed to sit in a comfortable chair and to rest their chins on the chin rest. The experiment started with 14 practice trials followed by the formal test. The formal test started with a nine-point calibration of eye-fixation position. During each trial, a black screen with a cross at the center was first presented for 1000 milliseconds in order to run a drift calibration. Next, a face photo was presented at the center of the screen. Participants were required to recognize the expression of each face photo and verbally report it as quickly and accurately as possible. The photo stayed on the screen until the participant's verbal report was detected by the voice response box, which in turn, triggered the next trial. The eye movements and response time were automatically recorded. The verbal reports were recorded by the experimenter. The twenty-eight face photos were presented in a random order. The experiment lasted for about 40 minutes.

### 2.4. Data Reduction

Response accuracy was represented by the percent of correct responses (calculated by dividing the number of correct responses by the total number of trials for each participant under each emotional condition, ranging from 0-1. Response accuracy and response time were submitted to repeated measures analysis of variance (ANOVA) with Emotion (happy, neutral, surprise, disgust, sad, fear, angry) as within-subject factor and Group (the HD group and the LD group) as between-subject factor.

To further investigate how the two groups differed on their attention to facial features, four regions of interest (ROI) were defined for each facial photo: eyebrows (left and right), eyes (left and right), nose, and mouth. The ROIs were defined by tracing the borders and extending about 5% of the face width beyond the features using Eyelink data viewer software package. Dwell time in the ROI was computed by summing up the duration of all fixations contained in the ROI except the first fixation which was always located at the screen center. A repeated measures analysis of variance (ANOVA) was performed on dwell time with Emotion as within-subject factor and Group as between-subject factor.

In addition, to test whether the HD group paid more attention to the left visual hemifield than the LD group during recognition of facial expressions, asymmetric eye movements were calculated by subtracting the total dwell time in right visual hemifield from the total dwell time in left visual hemifield. The asymmetric eye movements were then submitted to a repeated measures analysis of variance (ANOVA) with Emotion and Visual hemifield (left and right) as within-subject factor, and Group as between-subject factor.

For all analyses, the alpha level was set at  .05. The Greenhouse–Geisser correction was applied when the assumption of sphericity was violated. The uncorrected degrees of freedom, epsilon values, and effect sizes (partial eta-squared) are reported. Paired *t*-tests were conducted to further examine main effects.

## 3. Results

**Figure 1:**
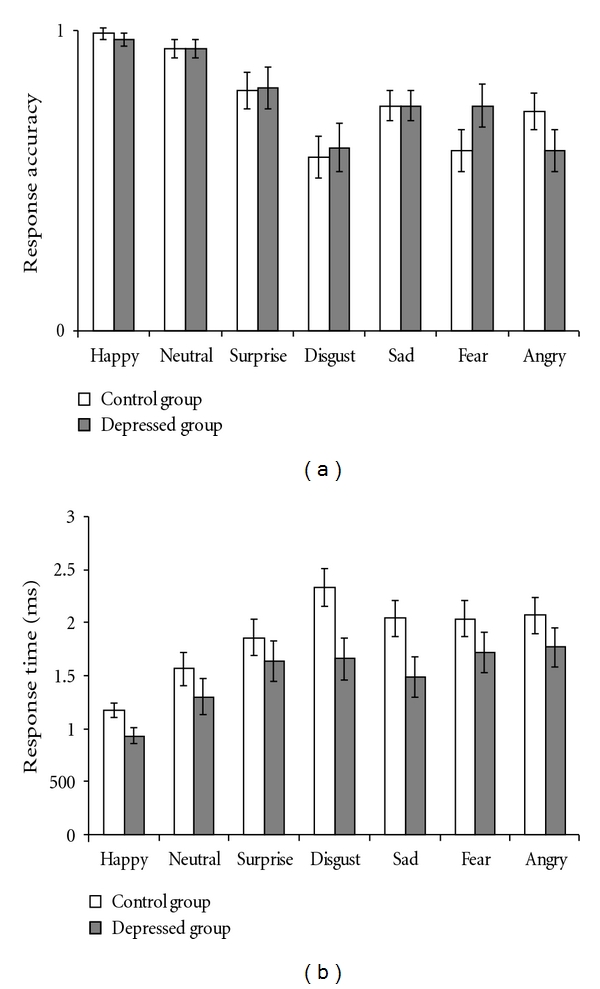
The means and standard error of response accuracy (i.e., percent of correct responses) (a) and response time (b) across each of the seven categories facial expression by the high- and low-depression groups.

**Figure 2:**
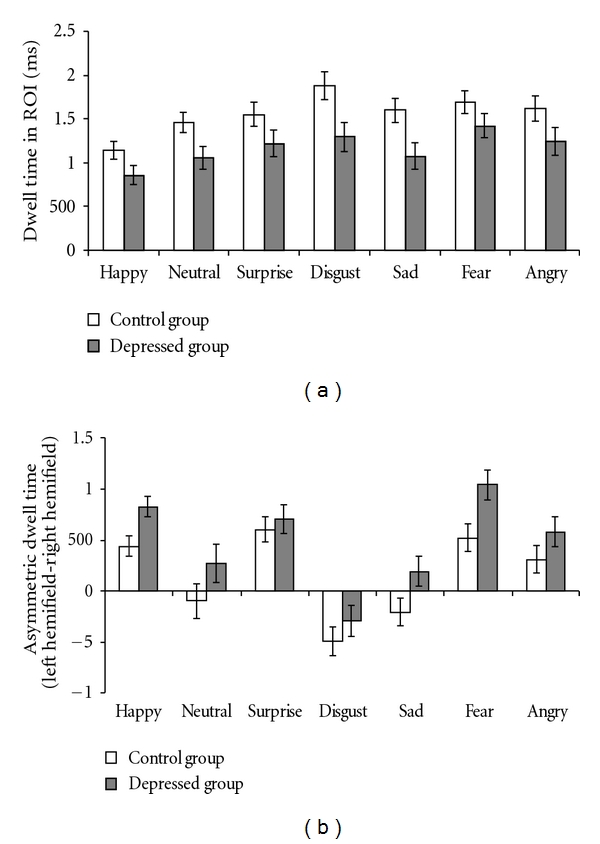
The means and standard error of dwell time in ROI (a) and asymmetric dwell time (b) across each of the seven categories facial expression by the high- and low-depression groups.

The means and standard error of verbal reports and eye movements to each facial expression by the HD group and the LD group are depicted in Figures 1 and 2, respectively.

### 3.1. Response Accuracy

There was a significant main effect of Emotion (*F*(6, 228) = 12.74, *P* < .001, partial *η* ^2^ = 0.25). Followup *t*-tests showed that, compared to response accuracy to neutral expressions, response accuracy to other emotional facial expressions was significantly lower (surprise: *t*(39) = −2.84, *P* < .01; disgust: *t*(39) = −6.05, *P* < .001; sad: *t*(39) = −4.78, *P* < .001; fear: *t*(39) = −4.93, *P* < .001; angry: *t*(39) = −5.28, *P* < .001) except happy expressions (*t*(39) = 1.36, *P* = 0.18). The main effect of Group and the interaction of Group and Emotion were not significant (*Fs* < 1, *ps* = *ns*).

### 3.2. Response Time (RT)

The ANOVA revealed a significant main effect of Group (*F*  (1, 38) = 4.67, *P* < .05, partial *η* ^2^ = 0.11) indicating that the HD group recognized facial expressions faster than the LD group. There was also a significant main effect of Emotion (*F*  (6, 228) = 13.40, *P* < .001, partial *η* ^2^ = 0.26). Followup *t*-tests showed that, compared to the RT to neutral expression, participants responded faster to happy expression (*t*(39) = −3.38, *P* < .01), but slower to all other facial expressions (surprise: *t*(39) = 2.37, *P* < .05; disgust:  *t*(39) = 3.77, *P* < .01; sad: *t*(39) = 2.82, *P* < .01; fear: *t*(39) = 3.20, *P* < .01; angry:  *t*(39) = 3.14, *P* < .01). The interactive effect of Group and Emotion was not significant (*F*  (6, 228) = 0.93, *P* = .48, partial *η* ^2^ = 0.02).

### 3.3. Eye Movements

#### 3.3.1. Dwell Time

The ANOVA indicated significant main effect of Group (*F*  (1, 38) = 7.96, *P* < .01, partial *η* ^2^ = 0.17); the HD group spent less time on ROIs than the LD group. There was also a significant main effect of Emotion (*F*  (6, 228) = 6.80, *P* < .001, partial *η* ^2^ = 0.15). Followup *t*-tests showed that, compared to dwell time on neutral expression, dwell time on happy expressions was significantly shorter (*t*  (39) = −3.22, *P* < .01), while dwell time on other expressions was significantly longer (disgust: *t*(39) = 3.15, *P* < .01; *P* = .01; fear: *t*(39) = 2.70, *P* = .01) except for surprise, sad, and angry, all *t* < 1.6, *ps* = *ns*. The interactive effect of Group and Emotion was not significant (*F*  (6, 228) = 0.61, *P* = .70, partial *η* ^2^ = 0.02). An additional repeated measures ANOVA was also conducted to test the dwell time differences between 

ROIs. ^1^

#### 3.3.2. Asymmetric Eye Movements

The ANOVA revealed a significant main effect of Group (*F*  (1, 38) = 4.64, *P* < .05, partial *η* ^2^ = 0.11) indicating that the HD group showed greater left bias to facial expressions than the LD group. There was also a significant main effect of Emotion (*F*  (6, 228) = 34.56, *P* < .001, partial *η* ^2^ = 0.48), whereas the interaction of Group and Emotion was not significant (*F*  (6, 228) = 0.92, *P* = .47, partial *η* ^2^ = 0.02). Followup *t*-tests showed that, compared to neutral expression, participants showed significantly greater left bias to all emotional facial expressions (happy: *t*(39) = 4.54, *P* < .001; surprise: *t*(39) = 5.47, *P* < .001; fear: *t*(39) = 6.22, *P* < .001; anger: *t*(39) = 3.03, *P* = .004; except for disgust (*t*(39) = −  3.61, *P* = .001) and sad facial expressions (*t*(39) = 0.78, *P* = .44).

## 4. Discussion

This study aimed to examine the differences between individuals with high and low depressive symptoms on facial expression recognition and to explore the cognitive systems underlying those differences. The main results indicate that the HD group responded much quicker to all facial expressions than the LD group, while performing as accurately as the LD group on facial expression recognition, suggesting an enhanced ability to recognize facial expressions. Moreover, this study revealed an abnormal eye-movement pattern in depression. Compared to the LD group, the HD group dwelled less on ROI in general and spent more time viewing the left visual hemifield of facial expressions than the right visual hemifield.

Our finding of an enhanced ability to recognize all categories of facial expressions in the HD group is consistent with previous studies that observed better performance on facial expression recognition task in dysphoric individuals [6], clinically diagnosed depressed patients [24], as well as remitted depressed individuals [5] than in controls. However, other studies reported specific or general deficits in facial expression recognition in depression [10, 25]. A reason for this inconsistency may be that the various tasks used in previous studies involved different cognitive processes such as selective attention and memory [13]. Thus, the deficits observed in those tasks may not characterize impaired ability to decode facial expressions, but potentially higher-order cognitive processes (e.g., selective attention and memory) which are affected by negative mood [6]. Compared to prior work, this study adopted a more direct and sensitive task which requires participants to verbally label facial expression by relying primarily on emotional decoding of facial expressions and thus may lead to a more solid conclusion on the abnormal facial expression recognition in depression individuals.

This study also observed abnormal eye-movement patterns in the HD group during facial expression recognition. In particular, individuals with elevated levels of depressive symptoms dwelled significantly less time than the LD group on regions of interest (ROI) including eyebrows, eyes, nose, and mouth. This is consistent with our findings of faster response times to facial expressions in the HD groups.

Importantly, this study also revealed greater left-side-biased eye movements in individuals with elevated levels of depressive symptoms, compared to the LD group. All participants dwelled longer on the part of the face in the left compared to the right visual hemifield, however, individuals with elevated levels of depressive symptoms showed even greater left-side preference across all categories of facial expressions. This left-side-bias effect has been argued to be an indicator of right hemisphere involvement in the processing of facial expressions [26, 27]. Evidence from EEG consistently reported larger activation in the right hemisphere compared with the left hemishphere during processing of emotional and facial relevant stimuli [28, 29]. Recent fMRI studies further localized the EEG asymmetries to specific areas of prefrontal cortex. In particular, it is confirmed that emotional stimulus processing and trait depression are associated with increased right-lateralized activation in subregions of the the dorsolateral prefrontal cortex (DLPFC) [30, 31]. Therefore, the greater left-side bias found in the HD group indicates hyperactivation in the right hemisphere and hence enhanced sensitivity to facial expressions, which may explain the fast response times without reduced accuracy. It would be interesting for future studies to explore the casual relation of depressive symptoms and this abnormal eye-movement pattern.

Consistent with previous studies [17, 18, 32], our results indicate that all individuals dwelled most on the nose, less on the eyes and the mouth, and least on the eyebrows. This pattern of attention allocation may at least partially be due to the presentation mode: face photos were presented at the screen center with a dark background. Therefore, except for the eyes and mouth which may provide important clues for emotion recognition, the nose was the optimal position to focus upon because it was located at both the face center as well as the screen center. Conversely, the eyebrow area was the last position participants attended to, perhaps because it is the farthest ROI from the vision center.

Finally, no evidence was found for a negative bias in the HD group. This result is somewhat unexpected. According to cognitive and social interactive theories of depression, we predicted that individuals with elevated levels of depressive symptoms would recognize negative facial expressions better (i.e., more accurate and faster) than positive and neutral facial expressions. However, our results indicate that both the HD group and the LD group recognize happy and neutral facial expressions much quicker and more accurately than negative facial expressions. An explanation for this lack of a negative bias may be due to a ceiling effect, as both groups performed significantly better on positive and neutral facial expressions (94%–99% accuracy) than on other facial expressions. However, this cannot explain why the HD group responded quicker than the LD group while maintaining accuracy comparable to the LD group across all categories of facial expressions (including positive and neutral facial expressions). A plausible hypothesis is that the depressive related negative schema of self and others motivate an enhanced emotional processing, and hence lead to a global hypersensitivity to facial expressions as observed in the recognition task. In particular, the negative schema of self and others may activate more avoidant, less tolerant experience and behaviors, as well as higher levels of distress and fear when confronted with others' facial expression (e.g., [33]). Accordingly, depressed individuals could be more motivated to complete the recognition task by responding faster to face photos in order to shorten the exposure time to facial expressions.

In addition to these interesting findings, there are some limitations to this study. This study recruited undergraduate students with elevated levels of depressive symptoms rather than depressed patients, thus the findings may not generalize to a clinically depressed sample. Furthermore, the picture stimuli we used in this study have somewhat high intensities (mean intensity score is 6.6 on a 9-point scale). Future studies are needed to investigate how dysphoric and depressed individuals recognize ambiguous facial expressions.

In conclusion, this study reveals a relation between depressive symptoms and an enhanced ability to recognize facial expressions. Furthermore, this study identifies an underlying cognitive system that explains the enhanced ability exhibited by the high-depression group: greater leftward attention bias, presumably related to a right hemisphere hyper activation. This study expands previous studies on understanding the relation between visual attention processes and facial expression recognition in depression, which has an important implication for developing an attentional model of depression.

## Endnotes

1. Additional repeated measures ANOVA was conducted on dwell time in each ROI with ROI, Emotion as within-subject factor, and Group as between-subject factor. The results indicated a significant main effect of Emotion (*F*  (6, 228) = 7.07, *P* < .001, partial *η* ^2^ = 0.16) and a significant main effect of ROI (*F*  (3, 114) = 119.11,  *P* < .001, partial *η* ^2^ = 0.76), whereas the main effect of Group and all interactive effects were not significant (*F* < 3, *ps* = *ns*). The followup tests of within-subjects contrasts demonstrated that compared to dwell time on nose, dwell time on other regions (eyes, mouth, and brows) was significantly shorter (*Fs* > 90, *ps* < .001, partial *η* ^2^ > 0.70). Dwell time on either mouth or eyes was significantly longer than that on brows (*Fs* > 23, *ps* < .001, partial *η* ^2^ > 0.37). Also dwell time on eyes was significantly longer than that on the mouth (*F*  (1, 38) = 15.65, *ps* < .001, partial *η* ^2^ > 0.  29).
